# A comparative study on the efficacy of different combinational anti-seizure medication therapies following valproate monotherapy failure

**DOI:** 10.1186/s42494-025-00233-3

**Published:** 2025-10-14

**Authors:** Raowei Yan, Hesheng Zhang, Jia He, Wenyu Liu, Nanya Hao, Enhui Zhang, Yujie Chen, Zhujing Ou, Xintong Wu, Dong Zhou

**Affiliations:** 1 Department of Neurology, West China Hospital, Sichuan University, Chengdu, 610041 Sichuan China; 2 Department of Neurology, 363 Hospital, Chengdu, 610041 China

**Keywords:** Valproate, Anti-seizure medication, Add-on, Efficacy

## Abstract

**Background:**

Sodium valproate (VPA) is widely recognized as the first-line treatment for patients with epilepsy (PWE). However, current studies lack evidence to determine the best add-on medication following VPA monotherapy failure. Lamotrigine (LTG), levetiracetam (LEV), oxcarbazepine (OXC), topiramate (TPM), and carbamazepine (CBZ) also exhibit broad-spectrum activity for seizures. This study aims to compare the therapeutic efficacy of different anti-seizure medication combinations in PWE following valproate monotherapy failure.

**Methods:**

Individuals were categorized into five groups: VPA + LTG, VPA + LEV, VPA + TPM, VPA + OXC and VPA + CBZ. Each group was further subdivided based on seizure type: generalized onset, focal onset, or unknown onset. The effectiveness of these five groups was compared using variance, χ^2^ test and Kaplan–Meier survival analysis.

**Results:**

A total of 2656 PWEs were included in this study. The ≥ 50% response rates for subjects with generalized epilepsy when combining VPA with LTG, OXC, LEV, TPM, and CBZ were 89.6%, 81.0%, 77.9%, 77.7%, and 75.9%, respectively. The LTG group demonstrated significantly higher efficacy than the LEV, TPM, and CBZ groups (*P* < 0.05). The ≥ 50% response rate of LTG, OXC, LEV, TPM and CBZ for subjects with focal epilepsy were 86.3%, 88.9%, 79.3%, 75.9% and 74.8%, respectively; with the OXC group being significantly more effective than the LEV, TPM, and CBZ groups (*P* < 0.05).

**Conclusions:**

In this real-world study, we assessed the effectiveness of five anti-seizure medications as add-on therapy for PWE who failed sodium valproate monotherapy. Our findings suggest that combining LTG may be more effective for subjects with generalized epilepsy, while combining OXC may be more effective for subjects with focal epilepsy.

## Background

Epilepsy is a prevalent chronic neurological condition, second only to stroke, which is associated with high disability and long-term effects. Recognized as a neuropsychiatric disease by the World Health Organization, epilepsy significantly impacts both physical and mental health. Globally, over 65 million people are affected by epilepsy, with nearly 10 million patients with epilepsy (PWE) in China alone [1]. The substantial burden of epilepsy on individuals, their families, and society highlights the critical need for effective diagnostic and treatment strategies to manage recurrent seizures.

Currently, anti-seizure medications (ASMs) are the first-line treatment for epilepsy. Sodium valproate (VPA) is one of the most commonly prescribed ASMs in China, accounting for over 21% of all ASM prescriptions [2], and is widely accepted as the first choice for generalized onset seizures. VPA exhibits the ability to readily penetrate the blood–brain barrier, enhance the synthesis of gamma-aminobutyric acid (GABA), and inhibit its degradation, effectively reducing neuronal excitability and suppressing convulsions. Its broad-spectrum efficacy also makes it suitable for other types of epilepsy. However, only about 45.7% PWE can achieve seizure-free with monotherapy [3]. When monotherapy fails, combination therapy may increase the likelihood of seizure freedom [4]. However, the efficacy of combination therapy following VPA monotherapy failure has not been updated through research. Previous studies have demonstrated that specific regimens, such as VPA + topiramate (TPM), VPA + lamotrigine (LTG), VPA + levetiracetam (LEV) can enhance the antiepileptic effect [5–7]. Nevertheless, these studies lacked evidence on drug selections according to seizure type. Moreover, none of them were specifically designed to investigate the most effective drug combinations for achieving desired therapeutic outcomes following VPA monotherapy failure.

This study focuses on PWE who failed VPA monotherapy. In this study, we compared the efficacy of various combinations with VPA for different seizure types. Six ASMs (VPA, carbamazepine [CBZ], LEV, LTG, oxcarbazepine [OXC], TPM) included in the study are all broad-spectrum anti-seizure medications that frequently prescribed, accounting for over 80% of prescriptions [2]. LTG and LEV are recommended as first-line medications for generalized onset epilepsy or focal onset epilepsy concurrently. OXC and CBZ demonstrate effectiveness in subjects with focal epilepsy. Clinical data from PWE meeting the inclusion and exclusion criteria were collected at the neurology clinic of West China Hospital, Sichuan University. Stratified analysis was used to compare the clinical efficacy of VPA in combination with both new and traditional anti-seizure medications across various seizure types. This study aims to provide clinicians with a rational basis for selecting ASMs and offers new evidence-based insights for reducing ASM-related adverse reactions, enhancing PWE's quality of life, and improving clinical ASM efficacy.

## Methods

### Patient selection and group

In this study, we defined VPA monotherapy failure as recurrent seizures occurring within three times the longest preintervention inter-seizure interval, following administration of VPA monotherapy at maintenance doses exceeding 50% of the defined daily dose (DDD). Individuals were recruited from the Department of Neurology at West China Hospital between January 2009 and December 2019. Inclusion criteria were as follows: (I) PWE aged over 18 years old, diagnosed with epilepsy, and possessing complete clinical information; (II) PWE who had been receiving VPA as the initial first-line monotherapy at doses exceeding 50% of the defined daily dose; (III) seizure occur within three times preintervention inter-seizure interval before the initiation of add-on therapy; (IV) PWE who received one of the following drug combination therapies after the failure of VPA monotherapy: VPA + CBZ, VPA + LEV, VPA + LTG; VPA + OXC; VPA + TPM; (V) PWE were followed up for at least 1 year after initiating the two-drug combination therapy.

Exclusion criteria included: (I) PWE with severe heart, brain, liver, kidney insufficiency, malignant tumors, and mental disorders; (II) PWE who received the above-mentioned combination of two drugs for less than 1 year; (III) PWE with progressive lesions in central nervous system or intracranial tumors; (IV) PWE who did not complete treatment or could not be followed up regularly; (V) PWE who took a third ASM during the 1 year follow-up period.

The study was approved by the West China Hospital Medical Ethics Committee (No. 2019210).

### Design and outcome

Clinical and demographic data (gender, age, duration of disease, income, education, place of residence, compliance, side effects, drug costs, origin of seizures, etiology of epilepsy, comorbid conditions, and choice of drug regimens) were collected using a standardized questionnaire at baseline. Seizure frequency at baseline and during follow-up, as well as associated costs, were documented. Medication adherence report scale (MARS) was evaluated after 1-year follow-up. The use of antidepressants, sedatives, and proprietary Chinese medicines approved for epilepsy treatment was prohibited during the study.

The outcomes were as follows: primary outcome measures included ≥ 50% responder rates (percentage of subjects with a ≥ 50% reduction in seizure frequency during 1-year follow-up). Secondary outcome measures included ≥ 75% responder rates (percentage of subjects with a ≥ 75% reduction in seizure frequency during 1-year follow-up); seizure-free rates (percentage of seizure-free subjects at maintenance period); incidence of side effects (calculated based on subject reports), drug compliance (judged according to MARS) and annual average cost.

### Statistical methods and data analysis

Data were analyzed using SPSS 25.0 statistical software (IBM, Armonk, NY, USA). Missing data were handled using complete case analysis for baseline variables and validated through sensitivity analyses for outcome variables. Normally distributed measurement data were expressed as mean ± standard deviation, while count data were presented as rates or composition ratios. Baseline data of the five groups of ASMs regimens were analyzed using ANOVA or RxC table χ^2^ test. Stratified analysis was performed by dividing individuals into three subgroups: generalized onset, focal onset, and unknown onset epilepsy, in accordance with the 2017 ILAE classification. ANOVA or RxC table χ^2^ test was used to analyze the clinical efficacy of the five groups of ASM groups within each subgroup. Kaplan–Meier survival analysis was applied to compare the cumulative seizure-free rate at different stages for the five ASM groups within the two subgroups of generalized onset and focal onset. Results with a *P*-value less than 0.05 were considered statistically significant.

## Results

A total of 2656 subjects were included in this study, 1207 (45.5%) of whom were male. The mean age of the participants was 27.76 ± 14.72 years. All PWE had experienced treatment failure with valproate monotherapy, of which 652 (24.5%) received LTG as add-on therapy, 715 (26.9%) were treated with LEV as add-on therapy, 550 (20.7%) received OXC as add-on therapy, 387 (14.6%) were prescribed TPM as add-on therapy, and 352 (13.3%) received CBZ as add-on therapy. There were no significantly differences in the age, gender, marital status, disease duration, age of onset, place of residence, education, annual income, or employment status among subjects in the five different ASM regimen groups. In terms of seizure type, 1093 (41.2%) PWE had generalized seizures, 1144 (43.1%) PWE had focal seizures, and 419 (15.8%) PWE had seizures of unknown origin. Demographic data of subjects were shown in Table 1.

**Table 1:** Comparison of demographic baseline data of subjects in five groups

Clinical factors	VPA + LTG (652)	VPA + LEV (715)	VPA + OXC (550)	VPA + TPM (387)	VPA + CBZ (352)	*P*-value
Age (year)	27.63 ± 12.72	29.50 ± 14.39	21.41 ± 11.45	30.61 ± 17.47	31.25 ± 16.83	0.107
Sex (male/female)	301/351	345/370	236/314	144/243	181/171	0.098
Marriage (married/unmarried)	268/384	272/443	185/365	164/223	142/210	0.134
Married	268 (41.1%)	272 (38.1%)	185 (33.7%)	164 (42.7%)	142 (40.3%)	
Unmarried	384 (58.9%)	443 (61.9%)	365 (66.3%)	223 (57.3%)	210 (59.7%)	
Course (year)	6.25 ± 3.77	6.01 ± 3.80	8.97 ± 3.35	9.74 ± 5.12	10.21 ± 4.37	0.152
Residence						0.223
Urban	350 (53.9%)	463 (64.8%)	342 (62.2%)	203 (52.4%)	182 (51.7%)	
Rural	302 (46.1%)	252 (35.2%)	208 (37.8%)	184 (47.6%)	170 (48.3%)	
Education						0.109
Primary school or below	242 (37.2%)	280 (39.2%)	192 (34.9%)	165 (42.8%)	154 (43.9%)	
Middle school	268 (41.1%)	289 (40.5%)	235 (42.7%)	129 (33.5%)	111 (31.5%)	
University and above	142 (21.7%)	146 (20.3%)	123 (22.4%)	93 (23.7%)	87 (24.6%)	
Annual income (Yuan)						0.126
< 10000	152 (23.4%)	151 (21.1%)	135 (24.6%)	113 (29.3%)	98 (27.9%)	
10000–50000	418 (64.1%)	431 (60.3%)	319 (58.0%)	220 (56.9%)	216 (61.4%)	
> 50000	82 (12.5%)	133 (18.6%)	96 (17.4%)	54 (13.8%)	38 (10.7%)	
Work or not						0.195
Yes	226 (34.7%)	263 (36.8%)	159 (28.9%)	122 (31.6%)	145 (27.5%)	
No	426 (65.3%)	452 (63.2%)	391 (71.1%)	265 (68.4%)	207 (72.5%)	
Seizure type						0.032
Generalized onset	309 (47.4%)	317 (44.3%)	184 (33.5%)	175 (45.2%)	108 (30.7%)	
Focal onset	248 (38.0%)	309 (43.2%)	287 (52.2%)	137 (35.4%)	163 (46.3%)	
Unknown onset	95 (14.6%)	89 (12.5%)	79 (14.3%)	75 (19.4%)	81 (23.0%)	

*VPA* Sodium valproate, *LTG* Lamotrigine, *LEV* Levetiracetam, *OXC* Oxcarbazepine, *TPM* Topiramate, *CBZ* Carbamazepine

### Primary outcome: ≥ 50% response rate

**Fig. 1:**
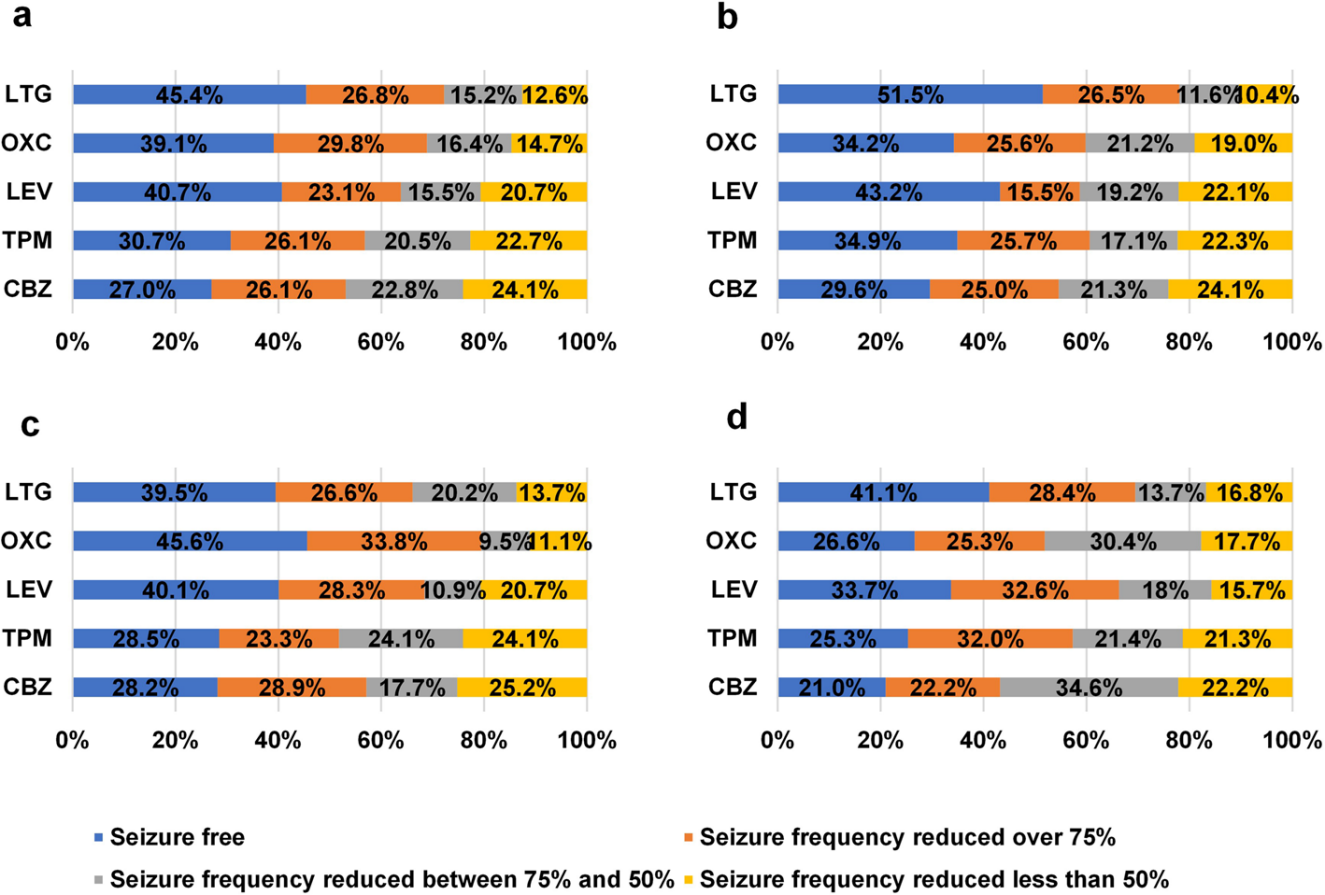
Treatment response of each subgroup. VPA: sodium valproate; LTG: lamotrigine; LEV: levetiracetam; OXC: oxcarbazepine; TPM: topiramate; CBZ: carbamazepine. a: treatment response of all included subjects. b: treatment response of subjects with generalized epilepsy; c: treatment response of subjects with focal epilepsy; d: treatment response of subjects with epilepsy of unknown origin

Treatment responses of PWE are presented in Fig. 1. The overall ≥ 50% response rate was 81.8%. The ≥ 50% response rate for LTG, OXC, LEV, TPM, and CBZ were 87.4%, 85.3%, 79.3%, 77.3%, and 75.9%, respectively. Pairwise comparisons indicate that the LTG group had a significantly higher response rate than the LEV, TPM, and CBZ groups. It was also higher than the OXC group, although this difference was not statistically significant.

Specifically, for different seizure types, the ≥ 50% response rates of LTG, OXC, LEV, TPM and CBZ for subjects with generalized epilepsy were 89.6%, 81.0%, 77.9%, 77.7%, and 75.9%, respectively. Pairwise comparisons indicate that the LTG group had a significantly higher response rate than the LEV, TPM, and CBZ groups. It was also higher than the OXC group, but this difference was not statistically significant.

The ≥ 50% response rates of LTG, OXC, LEV, TPM and CBZ for subjects with focal epilepsy were 86.3%, 88.9%, 79.3%, 75.9%, and 74.8%, respectively. Pairwise comparisons indicate that the OXC group had a significantly higher response rate than the LEV, TPM, and CBZ groups. It was also higher than the LTG group, although this difference was not statistically significant.

The ≥ 50% response rates of LTG, OXC, LEV, TPM and CBZ for subjects with epilepsy of unknown origin were 83.2%, 82.3%, 84.3%, 78.7%, and 77.8%, respectively. There were no significant differences among the groups. Table 2 shows the ≥ 50% response rates of different ASMs in different types of seizures.

**Table 2:** The ≥50% response rate of different ASMs in different types of seizures

	LTG	OXC	LEV	TPM	CBZ
Total	570 (87.4%) _a_	469 (85.3%) _a,b_	567 (79.3%) _b,c_	299 (77.3%) _c_	267 (75.9%) _c_
Generalized epilepsy	277 (89.6%) _a_	149 (81.0%) _a,b_	247 (77.9%) _b_	136 (77.7%) _b_	82 (75.9%) _b_
Focal epilepsy	214 (86.3%) _a,b_	255 (88.9%) _a_	245 (79.3%) _b,c_	104 (75.9%) _b,c_	122 (74.8%) _c_
Epilepsy of unknown origin	79 (83.2%)	65 (82.3%)	75 (84.3%)	59 (78.7%)	63 (77.8%)

*VPA* Sodium valproate, *LTG* Lamotrigine, *LEV* Levetiracetam, *OXC* Oxcarbazepine, *TPM* Topiramate, *CBZ* Carbamazepine

Same subscript indicates no statistical difference between the groups

### Secondary outcome: ≥ 75% response rate

The overall ≥ 75% response rate was 64.5%. The ≥ 75% response rates for LTG, OXC, LEV, TPM, and CBZ were 72.2%, 68.9%, 63.8%, 56.8%, and 53.1%, respectively. Pairwise comparisons indicate that the LTG group had a significantly higher response rate than the LEV, TPM, and CBZ groups. It was also higher than the OXC group, although this difference was not statistically significant.

Specifically, for different seizure types, the ≥ 75% response rates of LTG, OXC, LEV, TPM and CBZ for subjects with generalized epilepsy were 78.0%, 59.8%, 58.7%, 60.6%, and 54.6%, respectively. Pairwise comparisons indicate that the LTG group had a significantly higher response rate than the LEV, TPM, and CBZ groups. It was also higher than the OXC group, although this difference was not statistically significant.

The ≥ 75% response rates of LTG, OXC, LEV, TPM and CBZ for subjects with focal epilepsy were 66.1%, 79.4%, 68.3%, 51.8%, and 57.1%, respectively. Pairwise comparisons indicate that the OXC group had a significantly higher ≥ 75% response rate than the other groups.

The ≥ 75% response rates of LTG, OXC, LEV, TPM and CBZ for subjects with epilepsy of unknown origin were 69.5%, 51.9%, 66.3%, 57.3%, and 43.2%, respectively. The LTG group and LEV group had significantly higher response rates than the CBZ group. Table 3 shows the ≥ 75% response rates of different ASMs in different types of seizures.

**Table 3:** The ≥75% response rate of different ASMs in different types of seizures

	LTG	OXC	LEV	TPM	CBZ
Total	471 (72.2%) _a_	379 (68.9%) _a,b_	456 (63.8%) _b,c_	220 (56.8%) _c,d_	187 (53.1%) _d_
Generalized epilepsy	309 (78.0%) _a_	184 (59.8%) _b_	317 (58.7%) _b_	175 (60.6%) _b_	108 (54.6%) _b_
Focal epilepsy	164 (66.1%) _b,c_	228 (79.4%) _a_	211 (68.3%) _b_	71 (51.8%) _c_	93 (57.1%) _b,c_
Epilepsy of unknown origin	66 (69.5%) _a_	41 (51.9%) _a,b_	59 (66.3%) _a_	43 (57.3%) _a,b_	35 (43.2%) _b_

*VPA* Sodium valproate, *LTG* Lamotrigine, *LEV* Levetiracetam, *OXC* Oxcarbazepine, *TPM* Topiramate, *CBZ* Carbamazepine

Same subscript indicates no statistical difference between the groups

### Secondary outcome: seizure-free rate

A total of 1016 (38.3%) subjects achieved seizure freedom. The seizure-free rates in the LTG, OXC, LEV, TPM, and CBZ groups were 45.4%, 39.1%, 40.7%, 30.7%, and 27.0%, respectively. A χ^2^ test suggested that the LTG and LEV groups had significantly higher seizure-free rates than the TPM and CBZ groups. There were no significant differences among the LTG, LEV, and OXC groups.

In subjects with generalized epilepsy, the seizure-free rates in the LTG, OXC, LEV, TPM, and CBZ groups were 51.5%, 34.2%, 43.2%, 34.9%, and 29.6%, respectively. A χ^2^ test indicated that LTG was significantly higher than OXC, TPM, and CBZ groups, and was also higher than the LEV group, although not significantly.

In subjects with focal epilepsy, the seizure-free rates in the LTG, OXC, LEV, TPM, and CBZ groups were 39.5%, 45.6%, 40.1%, 28.5%, and 28.2%, respectively. The χ^2^ test suggested that OXC was significantly higher than the TPM and CBZ groups, and was also higher than the LTG and LEV groups, although not significantly.

In subjects with epilepsy of unknown origin, the seizure-free rates in the LTG, OXC, LEV, TPM, and CBZ groups were 41.1%, 26.6%, 33.7%, 25.3%, and 21.0%, respectively. The χ^2^ test indicated that LTG was significantly higher than the CBZ group and higher than the other three groups, although not significantly. Table 4 provides details on the seizure-free rate of different ASMs in different types of seizures.

**Table 4:** Seizure-free rate of different ASMs in different types of seizures

	LTG	OXC	LEV	TPM	CBZ
Total	296 (45.4%) _a_	215 (39.1%) _a,b_	291 (40.7%) _a_	119 (30.7%) _b,c_	95 (27.0%) _c_
Generalized epilepsy	159 (51.5%) _a_	63 (34.2%) _b_	137 (43.2%) _a,b_	61 (34.9%) _b_	32 (29.6%) _b_
Focal epilepsy	98 (39.5%) _a,b_	131 (45.6%) _a_	124 (40.1%) _a,b_	39 (28.5%) _b_	46 (28.2%) _b_
Epilepsy of unknown origin	39 (41.1%) _a_	21 (26.6%) _a,b_	30 (33.7%) _a,b_	19 (25.3%) _a,b_	17 (21.0%) _b_

*VPA* Sodium valproate, *LTG* Lamotrigine, *LEV* Levetiracetam, *OXC* Oxcarbazepine, *TPM* Topiramate, *CBZ* Carbamazepine

Same subscript indicates no statistical difference between the groups

**Fig. 2:**
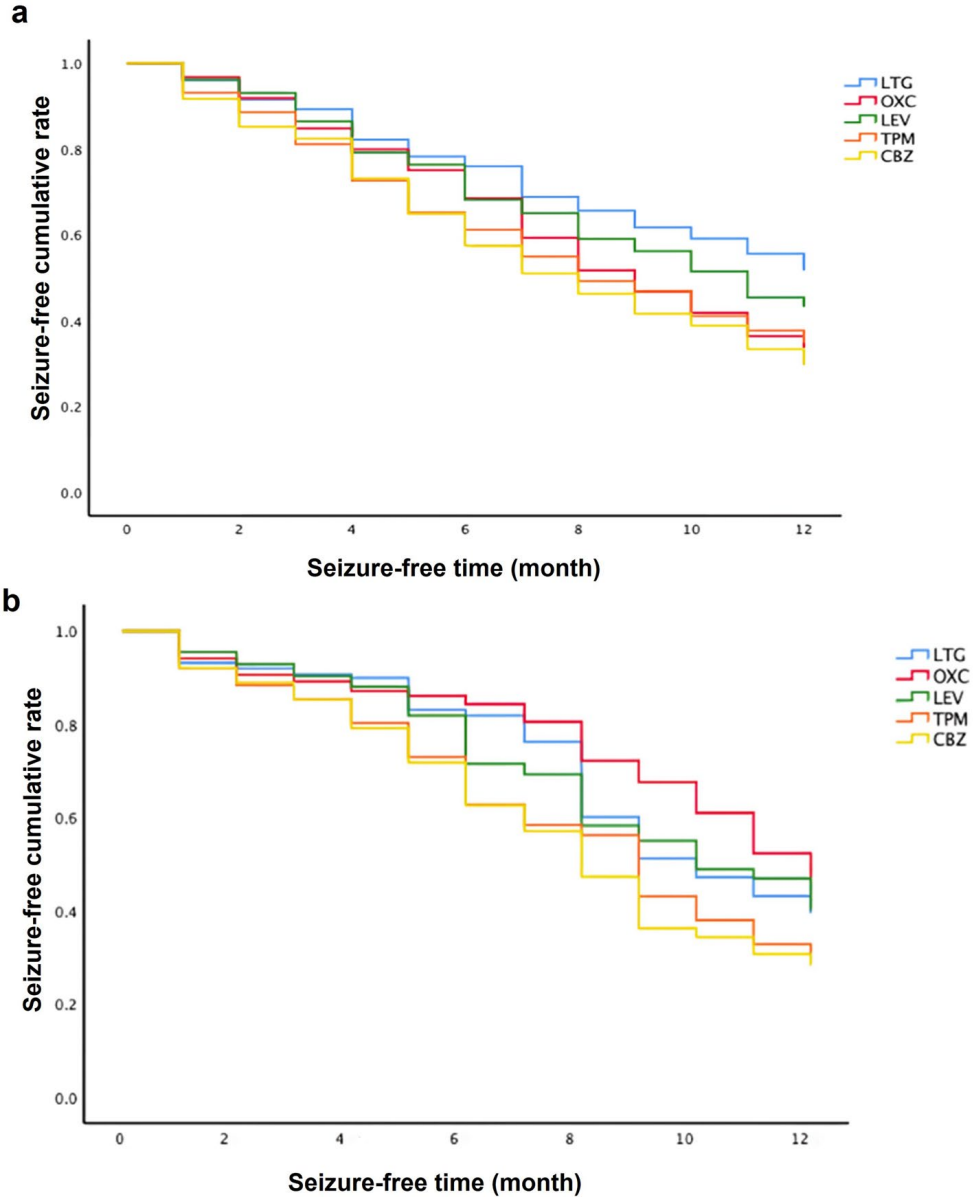
Kaplan–Meier curve for included individuals. VPA: Sodium valproate; LTG: Lamotrigine; LEV: Levetiracetam; OXC: Oxcarbazepine; TPM: Topiramate; CBZ: Carbamazepine. a: Kaplan–Meier of subjects with generalized epilepsy; b: Kaplan–Meier of subjects with focal epilepsy. Seizure-free time: the duration from the initiation of the add-on therapy to the first seizure recurrence

Kaplan–Meier curves for subjects are shown in Fig. 2. The log-rank test indicates that there is a significant difference between the LTG group and each of the other groups for subjects with generalized epilepsy (LTG *P* < 0.001, LEV *P* = 0.039, TPM *P* < 0.001, CBZ *P* < 0.001), and a significant difference between the OXC group and each of the other groups for subjects with focal epilepsy (LTG *P* = 0.022, LEV *P* = 0.003, TPM *P* < 0.001, CBZ *P* < 0.001).

### Secondary outcome: compliance

The distribution of compliance in each subgroup is shown in Table 5. There was no significant difference in compliance among the five groups, and there was no significant difference in compliance among the five treatment methods in each subgroup.

**Table 5:** Distribution of compliance

		LTG	LEV	OXC	TPM	CBZ	*P*
General epilepsy	Poor	21 (7.0%)	28 (8.8%)	32 (17.4%)	26 (14.9%)	39 (36.1%)	0.083
	Moderate	97 (31.4%)	92 (29.0%)	48 (26.1%)	60 (34.3%)	15 (13.9%)	
	Good	191 (61.6%)	197 (62.2%)	104 (56.5%)	89 (50.8%)	54 (50.0%)	
Focal epilepsy	Poor	17 (6.9%)	11 (3.6%)	21 (7.3%)	20 (14.6%)	24 (14.7%)	0.126
	Moderate	105 (42.3%)	135 (43.7%)	123 (42.9%)	52 (38.0%)	73 (44.8%)	
	Good	126 (50.8%)	163 (52.8%)	143 (49.8%)	65 (47.4%)	66 (40.5%)	
Epilepsy of unknown origin	Poor	11 (11.6%)	13 (14.6%)	17 (21.5%)	19 (25.3%)	21 (25.9%)	0.124
	Moderate	37 (38.9%)	39 (43.8%)	21 (26.6%)	24 (32.0%)	23 (28.4%)	
	Good	47 (49.5%)	37 (41.6%)	41 (51.9%)	32 (42.7%)	37 (45.7%)	

Compliance categories were defined as: Poor (MARS < 15), Moderate (MARS 15–19), Good (MARS 20–25). MARS: Medication Adherence Report Scale

*VPA* Sodium valproate, *LTG* Lamotrigine, *LEV* Levetiracetam, *OXC* Oxcarbazepine, *TPM* Topiramate, *CBZ* Carbamazepine

### Secondary outcome: side effects

The rate of side effects in each subgroup is shown in Table 6. There was no significant difference in rate of side effects among the five groups, and there was no significant difference in rate of side effects among the five treatment methods in each subgroup. The most frequently reported adverse effects across all treatment groups included sleepiness, dizziness, tiredness and headache. A detailed breakdown by treatment group is provided in Table 7.

**Table 6:** Side effects in each subgroup

	LTG	LEV	OXC	TPM	CBZ	*P*
General epilepsy	95 (30.7%)	72 (22.7%)	53 (28.8%)	38 (21.7%)	45 (41.7%)	0.112
Focal epilepsy	87 (35.1%)	98 (31.7%)	113 (39.4%)	51 (37.2%)	73 (44.8%)	0.145
Epilepsy of unknown origin	27 (28.4%)	20 (22.4%)	31 (39.2%)	29 (38.7%)	39 (48.1%)	0.102

*VPA* Sodium valproate, *LTG* Lamotrigine, *LEV* Levetiracetam, *OXC* Oxcarbazepine, *TPM* Topiramate, *CBZ* Carbamazepine

**Table 7:** Common side effects in each group

	LTG	LEV	OXC	TPM	CBZ
Tiredness	21 (3.2%)	28 (3.9%)	24 (4.4%)	21 (5.4%)	17 (4.8%)
Feeling of anger/aggressive	14 (2.1%)	23 (3.2%)	6 (1.1%)	8 (2.1%)	5 (1.4%)
Headache	19 (2.9%)	18 (2.5%)	28 (5.1%)	6 (1.6%)	19 (5.4%)
Rash	37 (5.7%)	5 (0.7%)	3 (0.5%)	1 (0.3%)	9 (2.6%)
Blurred vision	8 (1.2%)	8 (1.1%)	23 (4.2%)	4 (1.0%)	17 (4.8%)
Upset stomach	17 (2.6%)	15 (2.1%)	27 (4.9%)	6 (1.6%)	18 (5.1%)
Difficulty concentrating	9 (1.4%)	13 (1.8%)	11 (2.0%)	5 (1.3%)	6 (1.7%)
Shaky hands	16 (2.5%)	6 (0.8%)	13 (2.4%)	7 (1.8%)	11 (3.1%)
Dizziness	21 (3.2%)	26 (3.6%)	23 (4.2%)	22 (5.7%)	24 (6.8%)
Sleepiness	18 (2.8%)	35 (4.9%)	24 (4.4%)	25 (6.5%)	19 (5.4%)
Depression	7 (1.1%)	3 (0.4%)	4 (0.7%)	4 (1.0%)	2 (0.6%)
Memory problem	8 (1.2%)	5 (0.7%)	3 (0.5%)	3 (0.8%)	4 (1.1%)
Others	14 (2.1%)	5 (0.7%)	8 (1.5%)	6 (1.6%)	6 (1.7%)

*VPA* Sodium valproate, *LTG* Lamotrigine, *LEV* Levetiracetam, *OXC* Oxcarbazepine, *TPM* Topiramate, *CBZ* Carbamazepine

### Secondary outcome: cost

The annual average cost in each subgroup is shown in Table 8. Annual average cost of LEV was significantly higher than other groups in all three subgroups.

**Table 8:** Annual average cost in each subgroup

	LTG	LEV	OXC	TPM	CBZ	*P*
General epilepsy	43.66 ± 23.0	78.79 ± 36.7	61.3 ± 30.7	39.5 ± 16.3	31.6 ± 14.3	0.032
Focal epilepsy	46.5 ± 12.8	81.7 ± 24.1	60.5 ± 16.3	38.9 ± 15.8	33.4 ± 16.2	0.025
Epilepsy of unknown origin	44.52 ± 21.7	82.57 ± 41.2	65.9 ± 32.3	42.6 ± 20.7	34.9 ± 17.4	0.025

*VPA* Sodium valproate, *LTG* Lamotrigine, *LEV* Levetiracetam, *OXC* Oxcarbazepine, *TPM* Topiramate, *CBZ* Carbamazepine

Annual average cost is expressed in hundred Chinese Yuan (CNY)

## Discussion

In this study, we found that among the five anti-seizure medications (LTG, OXC, LEV, TPM, and CBZ), for PWE who had experienced VPA monotherapy failure, LTG group exhibited higher response rates (≥ 75% and ≥ 50%) and a higher seizure-free rate in subjects with generalized epilepsy. In contrast, for subjects with focal epilepsy, OXC group demonstrated superior response rates (≥ 75% and ≥ 50%) and a higher seizure-free rate. Notably, there were no significant differences in side effects or compliance among these five drugs, although LEV incurred higher costs.

In China, VPA is frequently employed as a first-line anti-seizure medication. However, when PWE do not respond well to VPA therapy, the option of add-on therapy becomes variable. In this study, 54.5% of the enrolled subjects were female, which represents a higher proportion compared to males. Previous studies have shown that VPA users were predominantly male [8, 9]. The use of VPA by pregnant women has been associated with birth defects, cognitive deficits, and an increased risk of developmental disabilities in the fetus, as confirmed by numerous studies [10–13]. Many guidelines now no longer recommend the use of VPA in women of reproductive age. This study included a broad range of patient ages, encompassing many non-reproductive-age female subjects, which may explain the higher proportion of female subjects. Additionally, due to the extended time span of this study, a consensus on avoiding VPA had not yet been reached in early stages. A previous study [2] showed that VPA was the most commonly used anti-seizure medication among adult women in China before 2015. However, since 2016, with the recommendation in guidelines to avoid the use of VPA in women of childbearing age and in pediatric female subjects [14], the use of VPA in adult women in China has steadily declined, with LEV replacing it as the most commonly used anti-seizure medication. This trend aligns with similar studies conducted in other countries [15–17], indicating that Chinese doctors are adopting a more cautious approach to the use of VPA in women, particularly those of childbearing age, in accordance with the guidelines.

The five drugs chosen for this study are commonly used as adjunctive therapies. Lamotrigine has been used as both monotherapy or adjunctive therapy for focal epilepsy, primary generalized tonic–clonic seizures, and Lennox-Gastaut syndrome. Several studies have demonstrated that combining LTG with VPA is more effective than using it as monotherapy or in other combinations. Brodie et al. [18] found that LTG as adjunctive therapy had good effects in PWE who failed treatment with VPA monotherapy, compared with PWE treated with CBZ and phenytoin monotherapy. Moeller et al. [6]. reported 35 cases of PWE with refractory epilepsy who failed treatment with an average of five ASMs. Among these, 18 cases achieved seizure freedom, and 4 cases showed some improvement with LTG-VPA therapy. Notably, 11 of the 22 subjects who experienced improvement had previously failed to respond to LTG and VPA monotherapy. Poolos et al.'s study [19] found that among 32 commonly used combinations of anti-seizure medications, the combination of LTG and VPA had the best seizure control efficacy; and further study showed improved response with escalation of LTG dosage [20]. In large cohort studies, Lee et al. [21] reported a seizure-free rate of 64.1% for the LTG + VPA combination at a 60-months follow-up, significantly higher than CBZ monotherapy. These findings have been consistently supported by numerous subsequent studies [22, 23], confirming the priority of LTG as an adjunctive therapy of valproate. In our study, the seizure-free rate in the LTG group was 45.4% at 12-month follow-up, slightly lower than Lee's research [21]. This inconsistency may stem from the differences in the subjects. It's important to note that they used combination therapy for subjects with new-onset epilepsy. However, our study included PWE experienced VPA monotherapy failure, who may represent a more refractory group. Additionally, the shorter follow-up period (1 year vs. 5 years) in our study may underestimate long-term efficacy. Specifically, for subjects with generalized epilepsy, LTG provided better results compared to the other four drugs with 50% response rate, 75% response rate, and seizure-free rate of 89.6%, 78.0%, and 51.5%, respectively, at 12-month follow-up.

Pharmacological studies have provided further evidence of the synergistic effect between VPA and LTG. Rowland et al. [24] conducted an in vitro pharmacokinetic study revealing that valproic acid inhibits the metabolism of LTG by affecting N2-glucuronidation of LTG through the UDP-glucuronosyltransferase 2B7 peptide family. Numerous pharmacokinetic studies confirmed that VPA can significantly reduce the mean clearance rate of LTG, thereby extending its half-life, and increasing its blood concentration in either PWE or healthy subjects [25–27]. It's also worth noting that the use of LTG in combination therapy may enhance the risk of adverse reactions, such as rashes. Therefore, careful monitoring for such potential side effects should be an integral part of the treatment plan [28].

Oxcarbazepine, another widely used voltage-gated sodium channel blocker, can be used as either monotherapy or adjunctive therapy for the treatment of focal or generalized tonic–clonic seizures, acting through its 10-hydroxy metabolite [29]. Studies have indicated a positive correlation between the antiepileptic efficacy of OXC and the concentration of 10-hydroxy CBZ in the bloodstream. Furthermore, valproic acid can displace 10-hydroxy CBZ from its plasma protein binding site, thereby enhancing the antiepileptic effect of OXC, and exerting a synergistic effect in the treatment of epilepsy. Peng et al. [30] demonstrated that OXC in combination with VPA was more effective than monotherapy and that this advantage was more significant in subjects with focal epilepsy, consistent with the results of our study. In our study, among subjects with refractory focal epilepsy who failed monotherapy with VPA, OXC, as an adjunctive treatment, demonstrated a higher 75% response rate compared to LTG, LEV, TPM, and CBZ. It also showed a higher 50% response rate compared to LEV, TPM, and CBZ, as well as a higher seizure-free rate compared to TPM and CBZ.

This study serves as a foundation for the development of anti-epileptic treatment protocols and offers valuable guidance for the rational clinical use of medications, underscoring its significant clinical implications. For patients with generalized epilepsy, VPA + LTG demonstrated superior efficacy, suggesting it should be prioritized in this subgroup. Conversely, VPA + OXC achieved the highest response rates in focal epilepsy, supporting its use as a first-line adjunctive option. However, it's essential to acknowledge that this study is a retrospective cohort analysis with a large sample size and numerous confounding factors. Clinician preferences in drug selection and patient biases, influenced by factors like drug cost, accessibility, education level, and adherence, may have played a role in the study outcomes. Meanwhile, several limitations exist in this study. Firstly, although our sensitivity analyses suggested minimal bias from missing data, the use of complete case analysis may underestimate variability in subgroups with higher missing rates. Secondly, the study had a relatively short 1-year follow-up period, which might have limited the ability to observe higher response rates over a more extended duration. Additionally, the absence of etiological data limited the generalizability of our findings. Patients with different underlying causes of epilepsy may exhibit varied responses to combination therapies, and this could not be explored in the current study. Furthermore, in recent years, newer anti-seizure medications such as perampanel, lacosamide, and zonisamide have gained wider acceptance and use. Further research could incorporate these drugs to provide more comprehensive evidence for the choice of add-on therapy. In the future, researches comparing VPA + LTG and VPA + OXC with newer broad-spectrum ASMs are needed to validate our findings in generalized and focal epilepsy subgroups, respectively. In addition, extended follow-up studies could be conducted to assess the sustainability of seizure freedom, development of drug resistance, and long-term adverse effects associated with combination therapies.

## Conclusions

In conclusion, in this real-world study, we compared the effectiveness of five anti-seizure medications as add-on therapy for PWE who failed VPA monotherapy and found that LTG may be more effective in subjects with generalized epilepsy and OXC may be more effective in subjects with focal epilepsy.

## Data Availability

The data that support the findings of this study are available from the corresponding author upon reasonable request.

## Abbreviations

ASM: Anti-seizure medication
CBZ: Carbamazepine
LEV: Levetiracetam
LTG: Lamotrigine
OXC: Oxcarbazepine
TPM: Topiramate
VPA: Valproate

## References

[1] Song P, Liu Y, Yu X, Wu J, Poon AN, Demaio A, et al. Prevalence of epilepsy in China between 1990 and 2015: a systematic review and meta-analysis. J Glob Health. 2017;7(2):020706. 29302325 10.7189/jogh.07-020706 PMC5737100

[2] Yu L, Zhu W, Zhu X, Lu Y, Yu Z, Dai H. Anti-seizure medication prescription in adult outpatients with epilepsy in China, 2013–2018. Front Neurol. 2021;12:649589. 34108928 10.3389/fneur.2021.649589 PMC8180859

[3] Chen Z, Brodie MJ, Liew D, Kwan P. Treatment outcomes in patients with newly diagnosed epilepsy treated with established and new antiepileptic drugs: a 30-year longitudinal cohort study. JAMA Neurol. 2018;75(3):279–86. 29279892 10.1001/jamaneurol.2017.3949 PMC5885858

[4] Chi X, Li R, Hao X, Chen J, Xiong W, Xu H, et al. Response to treatment schedules after the first antiepileptic drug failed. Epilepsia. 2018;59(11):2118–24. 30246334 10.1111/epi.14565

[5] Ji ZY, Huang YQ, He WZ. Sodium valproate combined with topiramate vs. sodium valproate alone for refractory epilepsy: a systematic review and meta-analysis. Front Neurol. 2021;12:794856. 35069424 10.3389/fneur.2021.794856 PMC8766331

[6] Moeller JJ, Rahey SR, Sadler RM. Lamotrigine-valproic acid combination therapy for medically refractory epilepsy. Epilepsia. 2009;50(3):475–9. 19054403 10.1111/j.1528-1167.2008.01866.x

[7] Metcalf CS, Gagangras S, Bulaj G, White HS. Synergistic effects of the galanin analog 810–2 with the antiseizure medication levetiracetam in rodent seizure models. Epilepsia. 2022;63(12):3090–9. 36177529 10.1111/epi.17420

[8] Fujimoto A, Enoki H, Hatano K, Sato K, Okanishi T. Replacement of valproic acid with new anti-seizure medications in idiopathic generalized epilepsy. J Clin Med. 2022;11(15):4582. 35956197 10.3390/jcm11154582 PMC9369717

[9] Li J, Zhang X, Li N, Zhao D, Li G, Lin W. Mortality rates in people with convulsive epilepsy in rural Northeast China. Front Neurol. 2020;11:1013. 33041973 10.3389/fneur.2020.01013 PMC7517037

[10] Seshachala BB, Jose M, Lathikakumari AM, Murali S, Kumar AS, Thomas SV. Valproate usage in pregnancy: an audit from the Kerala registry of epilepsy and pregnancy. Epilepsia. 2021;62(5):1141–7. 33782943 10.1111/epi.16882

[11] Meador KJ, Baker GA, Browning N, Clayton-Smith J, Combs-Cantrell DT, Cohen M, et al. Cognitive function at 3 years of age after fetal exposure to antiepileptic drugs. N Engl J Med. 2009;360(16):1597–605. 19369666 10.1056/NEJMoa0803531 PMC2737185

[12] Cummings C, Stewart M, Stevenson M, Morrow J, Nelson J. Neurodevelopment of children exposed in utero to lamotrigine, sodium valproate and carbamazepine. Arch Dis Child. 2011;96(7):643–7. 21415043 10.1136/adc.2009.176990

[13] Baker GA, Bromley RL, Briggs M, Cheyne CP, Cohen MJ, Garcia-Finana M, et al. IQ at 6 years after in utero exposure to antiepileptic drugs: a controlled cohort study. Neurology. 2015;84(4):382–90. 25540307 10.1212/WNL.0000000000001182 PMC4336006

[14] Tomson T, Marson A, Boon P, Canevini MP, Covanis A, Gaily E, et al. Valproate in the treatment of epilepsy in girls and women of childbearing potential. Epilepsia. 2015;56(7):1006–19. 25851171 10.1111/epi.13021

[15] Ackers R, Besag FM, Wade A, Murray ML, Wong IC. Changing trends in antiepileptic drug prescribing in girls of child-bearing potential. Arch Dis Child. 2009;94(6):443–7. 19307197 10.1136/adc.2008.144386

[16] Virta LJ, Kalviainen R, Villikka K, Keranen T. Declining trend in valproate use in Finland among females of childbearing age in 2012–2016 - a nationwide registry-based outpatient study. Eur J Neurol. 2018;25(6):869–74. 29509301 10.1111/ene.13610

[17] Gaudio M, Konstantara E, Joy M, van Vlymen J, de Lusignan S. Valproate prescription to women of childbearing age in English primary care: repeated cross-sectional analyses and retrospective cohort study. BMC Pregnancy Childbirth. 2022;22(1):73. 35086478 10.1186/s12884-021-04351-x PMC8793222

[18] Brodie MJ, Yuen AW. Lamotrigine substitution study: evidence for synergism with sodium valproate? 105 study group. Epilepsy Res. 1997;26(3):423–32. 9127723 10.1016/s0920-1211(96)01007-8

[19] Poolos NP, Warner LN, Humphreys SZ, Williams S. Comparative efficacy of combination drug therapy in refractory epilepsy. Neurology. 2012;78(1):62–8. 22170887 10.1212/WNL.0b013e31823ed0dd

[20] Poolos NP, Castagna CE, Williams S, Miller AB, Story TJ. Association between antiepileptic drug dose and long-term response in patients with refractory epilepsy. Epilepsy Behav. 2017;69:59–68. 28235655 10.1016/j.yebeh.2016.10.010

[21] Lee BI, No SK, Yi SD, Lee HW, Kim OJ, Kim SH, et al. Unblinded, randomized multicenter trial comparing lamotrigine and valproate combination with controlled-release carbamazepine monotherapy as initial drug regimen in untreated epilepsy. Seizure. 2018;55:17–24. 29324401 10.1016/j.seizure.2017.12.008

[22] Pisani F, Oteri G, Russo MF, Di Perri R, Perucca E, Richens A. The efficacy of valproate-lamotrigine comedication in refractory complex partial seizures: evidence for a pharmacodynamic interaction. Epilepsia. 1999;40(8):1141–6. 10448829 10.1111/j.1528-1157.1999.tb00832.x

[23] McCabe PH, McNew CD, Michel NC. Effect of divalproex-lamotrigine combination therapy in frontal lobe seizures. Arch Neurol. 2001;58(8):1264–8. 11493167 10.1001/archneur.58.8.1264

[24] Rowland A, Elliot DJ, Williams JA, Mackenzie PI, Dickinson RG, Miners JO. In vitro characterization of lamotrigine N2-glucuronidation and the lamotrigine-valproic acid interaction. Drug Metab Dispos. 2006;34(6):1055–62. 16565174 10.1124/dmd.106.009340

[25] Xu S, Liu L, Chen Y, Liu M, Lu T, Wang H, et al. Population pharmacokinetics of lamotrigine co-administered with valproic acid in Chinese epileptic children using nonlinear mixed effects modeling. Eur J Clin Pharmacol. 2018;74(5):583–91. 29340733 10.1007/s00228-018-2414-8

[26] Eriksson AS, Hoppu K, Nergardh A, Boreus L. Pharmacokinetic interactions between lamotrigine and other antiepileptic drugs in children with intractable epilepsy. Epilepsia. 1996;37(8):769–73. 8764817 10.1111/j.1528-1157.1996.tb00650.x

[27] Anderson GD, Yau MK, Gidal BE, Harris SJ, Levy RH, Lai AA, et al. Bidirectional interaction of valproate and lamotrigine in healthy subjects. Clin Pharmacol Ther. 1996;60(2):145–56. 8823232 10.1016/S0009-9236(96)90130-7

[28] Reutens DC, Duncan JS, Patsalos PN. Disabling tremor after lamotrigine with sodium valproate. Lancet. 1993;342(8864):185–6. 8101290 10.1016/0140-6736(93)91398-6

[29] Wamil AW, Schmutz M, Portet C, Feldmann KF, McLean MJ. Effects of oxcarbazepine and 10-hydroxycarbamazepine on action potential firing and generalized seizures. Eur J Pharmacol. 1994;271(2–3):301–8. 7705430 10.1016/0014-2999(94)90787-0

[30] Peng Q, Ma M, Gu X, Hu Y, Zhou B. Evaluation of factors impacting the efficacy of single or combination therapies of valproic acid, carbamazepine, and oxcarbazepine: a longitudinal observation study. Front Pharmacol. 2021;12:641512. 34017250 10.3389/fphar.2021.641512 PMC8129194

